# HIV risk and pre‐exposure prophylaxis interest among women seeking post‐abortion care in Kenya: a cross‐sectional study

**DOI:** 10.1002/jia2.25703

**Published:** 2021-05-10

**Authors:** Renee Heffron, Edinah Casmir, Linda Aswani, Kenneth Ngure, Benn Kwach, Vallery Ogello, Catherine Kiptinness, Faith Ambiyo, Njeri Wairimu, Ethel Ossome, Hilda Machafu, Yasaman Zia, Dorothy Thomas, Caitlin Scoville, Taryn Barker, Elizabeth Bukusi, Nelly Mugo

**Affiliations:** ^1^ Department of Global Health University of Washington Seattle USA; ^2^ Department of Epidemiology University of Washington Seattle WA USA; ^3^ Partners in Health and Research Development Thika Kenya; ^4^ Center for Clinical Research Kenya Medical Research Institute Nairobi Kenya; ^5^ Center for Microbiology Research Kenya Medical Research Institute Nairobi Kenya; ^6^ Jomo Kenyatta University of Agriculture and Technology Nairobi Kenya; ^7^ Children’s Investment Fund Foundation London UK; ^8^ Department of Obstetrics and Gynecology University of Washington Seattle USA

**Keywords:** PrEP, abortion, Kenya, young women, HIV prevention

## Abstract

**Introduction:**

Post‐abortion clinics located in regions with high HIV burden may ideal locations to integrate counselling and delivery of HIV pre‐exposure prophylaxis (PrEP), aligning with normative goals for integrated delivery of HIV and reproductive health care. The objective of this study was to gauge the degree to which Kenyan women seeking care for a pregnancy loss, including induced abortion, are at risk for HIV and whether women would welcome an introduction to PrEP prior to discharge from post‐abortion care.

**Methods:**

We conducted a mixed‐methods study from August 2019 to February 2020 with women ages 15 to 30 recruited sequentially as they were accessing post‐abortion care at public and private facilities in Thika and Kisumu, Kenya. Data collection was through a cross‐sectional survey and laboratory testing for common sexually transmitted infections (N = 200), and in‐depth interviews (N = 30). Descriptive statistics summarize PrEP knowledge and referrals and a multivariable log‐link binomial model estimated correlates of receiving a referral for PrEP. Qualitative data were analysed using inductive and deductive approaches.

**Results:**

Among 200 HIV‐negative women (median age 21.0, interquartile range 19.0 to 22.0), the prevalence of *Chlamydia trachomatis* was 18.2% and *Neisseria gonorrhoeae* was 2.0%. Half of the women scored ≥5 on a validated tool that would correspond to an expected HIV incidence of 9.5% per year. Approximately half (55.8%) of women were familiar with PrEP prior to the study and 33.3% received a referral from study staff to a clinic offering PrEP. In qualitative interviews, women expressed interest in accessing PrEP from the gynaecology ward that provided post‐abortion care but they preferred alternative locations for PrEP refills.

**Conclusions:**

Kenyan women accessing post‐abortion care have substantial HIV risk and were favourable about the idea of receiving support to initiate PrEP as part of care offered during post‐abortion care. These settings can be integrated into national PrEP programmes as locations providing PrEP referrals and initiation.

## 1 INTRODUCTION

Young women living in settings with a high HIV burden face intersecting risks for unintended pregnancy, HIV and other sexually transmitted infections. To combat this, a priority strategy is to integrate programmes for reproductive health and HIV prevention, yielding provision of comprehensive care that can be delivered efficiently and in a cost‐saving manner [1]. Integration of HIV prevention, including delivery of pre‐exposure prophylaxis (PrEP), into existing reproductive health programmes has been demonstrated in clinics for family planning [2, 3, 4], antenatal and postnatal care [5], and safer conception [6, 7] and is facilitated by national guidelines in many settings that promote PrEP use during pregnancy [8]. When women in these settings have been offered PrEP as an additional routine service, PrEP uptake has been moderate overall (22% in the largest study) with the greatest uptake in women whose partners are known to be living with HIV (94%) [2]. Continuation rates at 6‐months appear low, mirroring observations of PrEP continuation among young women accessing PrEP as a stand‐alone service [9, 10].

Clinics providing abortion and post‐abortion care are one type of reproductive health service yet they are often overlooked for programmatic innovation due to the vulnerability of patients and the complicated legal situation of abortion. Kenya has the highest abortion rate in the East Africa region, estimated at 48/1000 women [11]. In a 2012 facility‐based survey, the peak age for induced abortion was estimated at 20 to 24 years (76 abortions/1000 women) with adolescents aged 15 to 19 years having a frequency of 38 abortions/1000 women [11]. Women living in settings with high HIV burden who access post‐abortion care may have a high risk for HIV and other sexually transmitted infections because condom use is low and likely to continue despite the recent unintended pregnancy [12, 13] A recent study in Kenya highlighted the frequency of repeated induced abortions among young women and unmet contraceptive need [11, 14], highlighting the potential for poor sexual and reproductive health outcomes among these women [14].

Post‐abortion clinics serve women with spontaneous and induced abortion who often need emergency care to be medically stabilized followed by treatment for complications and psychosocial and/or behavioural counselling [15]. Induced abortion is illegal in Kenya except to protect a woman’s life and health [16]. Thus, these clinics do not initiate abortions but they do provide life‐saving care and reduce the risk of maternal mortality. Currently in Kenya, 35% of maternal deaths are attributed to unsafe abortion [14, 17]. A routine component of post‐abortion care is counselling on contraceptive use and referral to family planning clinics. We postulate that this component of prevention counseling could be expanded to empower women to adopt effective HIV prevention strategies that are within their control, such as oral PrEP [18, 19]. In order to explore the potential to integrate PrEP into post‐abortion care, the objective of our study was to conduct formative work among young women seeking post‐abortion care in Kenya and estimate HIV risk and the potential for PrEP to be introduced.

## 2 METHODS

### 2.1 Study design and participants

We conducted a cross‐sectional mixed‐methods study in Thika and Kisumu, Kenya among women who were seeking care following a pregnancy loss. From August 2019 to February 2020, women seeking post‐abortion services were sequentially recruited by research staff who were contacted by nurses and clinical officers providing post‐abortion care at Thika sub‐County Hospital, KMET (a Kisumu‐based non‐governmental organization), and Jaramogi Oginga Odinga Teaching and Referral Hospital, when a potentially eligible women were seen at the facility. Eligibility criteria for these women included accessing care following a pregnancy loss and being 15 to 30 years. Women were excluded if living with HIV or with a social or physical condition that may make participation unsafe as determined by the study investigators. Enrolment was stopped once 200 women were accrued based on estimates of the sample size needed for moderate precision in estimates of STI prevalence.

All enrolled women completed a survey to characterize demographics, reproductive and medical history, sexual behaviour, relationship characteristics, use of alcohol and/or drugs, HIV risk perception and unintended pregnancy, experiences with the recent pregnancy and pregnancy loss, and knowledge of and experience with PrEP. The Awareness, Interest, Desire, Action marketing model was applied to experiences with PrEP to form a PrEP “knowledge‐to‐use” cascade. Heavy alcohol use was measured with the Rapid Alcohol Problem Screening tool [20] and all other items were measured with questions validated in recent PrEP and contraceptive studies [21, 22]. Women provided a blood sample for *Treponema pallidum* testing (with Rapid Plasma Reagin tests) and urine sample for *Neisseria gonorrhoeae* and *Chlamydia trachomatis* testing using Aptima GenProbe (for women enrolled in Thika) or GeneXpert (for women enrolled in Kisumu). Results from STI testing were returned to women via phone or in‐person based on participant preference; women with reactive results were given treatment according to national guidelines. If results from routinely conducted rapid HIV testing were available from the post‐abortion clinic, they were abstracted from clinic records and if they were not available, rapid HIV antibody testing was conducted by study staff and results were returned to women in real time. After completing the survey, women were counselled about PrEP and all women were offered a referral for PrEP services at a nearby facility of their choice. The woman’s preference for a referral/no referral was noted on the survey form. Research staff entered data from quantitative surveys and laboratory testing into a web‐based platform (CommCare).

A purposive sample of women who completed the survey and had a high risk for HIV was selected for in‐depth interviews. Drawing on standards for qualitative research, we aimed to enrol up to 30 women for this sub‐sample [23]. To determine HIV risk, an empirically derived score that includes items incorporating women’s age, marital status, alcohol use, partner provision of financial support, and whether the partner has additional sex partners was applied to each woman who completed the survey (i.e. the VOICE risk score with a range from zero to eight and we used a cutoff of >5 to denote high risk) [24]. A semi‐structured discussion guide was developed to elicit conversation about HIV and pregnancy risk, perceptions about contraception and PrEP, and experiences with care following a pregnancy loss. Qualitative interviews conducted in the participant’s preferred language (English, Kiswahili, or Dholuo) were recorded, transcribed and translated into English. If participants did not give permission for recording, detailed notes were taken in their place.

### 2.2 Analytic methods

Descriptive statistics were used to summarize participant characteristics, experiences with post‐abortion care, knowledge about PrEP, willingness to initiate PrEP in conjunction with post‐abortion care, and the frequency with which PrEP referrals were given. To identify characteristics more common among women who received a PrEP referral, we calculated relative risks using a log‐link binomial model; factors associated with PrEP referral at the level of *p* < 0.20 were included in a multivariable model. Qualitative interviews were analysed using inductive and deductive approaches [25]. An initial codebook was developed on questions in the guides and five randomly selected transcripts were read and coded in order to identify new codes. A team of 5 analysts (EC, VO, BK, DT, KN) grouped the codes into themes and broader topics and assigned definitions to each. The codebook with topics, themes, and codes was uploaded into Dedoose. During the coding process, new codes were added as they were identified and then applied to all transcripts. The analysts coded the transcripts independently: all coded the first transcript and came together to concur on their codes, then two people each coded the next three transcripts and came together to identify and adjust codes where discrepancies occurred. Finally, once coders were aligned in their understanding of codes, all remaining transcripts were distributed among coders and each was coded by one person. The team met bi‐weekly over Zoom to discuss progress and emerging themes. Dedoose v8.0 (Los Angeles, CA, USA) was used to facilitate the coding and analysis process and SAS 9.4 (Cary, NC, USA) was used for all quantitative analysis.

### 2.3 Ethics

The protocol was approved by the Scientific and Ethical Review Unit at the Kenya Medical Research Institute and the Human Subjects Division at the University of Washington. All participants completed written informed consent, including women ages 15 to 17 who were considered emancipated minors according to Kenya national research guidelines since they had recently been pregnant. Participants were reimbursed for their time and any expenses incurred (e.g. travel for interviews).

## 3 RESULTS

### 3.1 Participant characteristics

A total of two hundred women were enrolled for this study, including 30 who were included for in‐depth interviews (Table 1). Two women were excluded from analyses because they were discovered to be living with HIV after study enrolment. Women were a median age of 21 (interquartile range [IQR]: 19 to 22), with 13 years of schooling (IQR 11 to 14), and a median of 0 to 1 child living. While 39.4% reported being married (55% of women in Thika and 17.9% of women in Kisumu), 74.2% reported receiving financial or material support from a partner and 69.2% did not earn an income of their own. Applying the VOICE risk scoring tool for HIV risk, the median score was 5 (IQR: 3 to 6) overall which would be commensurate with an expected HIV incidence of 9.5% (95% CI 7.6% to 12.6%) per year [24]. Women reported a median of 2 (IQR 0 to 8) vaginal sex acts during the past month (Thika: 4, IQR 1 to 20) and Kisumu: 2, IQR 0 to 8) and condom use was relatively low with 11.6% reporting that a condom was used during the most recent vaginal sex and 95.7% of women reporting never, rarely, or sometimes using a condom during the past month. Most women had one partner in the past three months and this was a new partner for 9.1% of women. Most women reported that their partner was HIV negative (67.2%) or that they did not know their partner’s HIV status (32.3%) and 12.0% of women (6.4% in Thika and 22.9% in Kisumu) reported feeling obligated to have sex in exchange for money or goods. Twelve percent (12.2%) of women reported using alcohol and half of those using alcohol indicated heavy use. The prevalence of *Neisseria gonorrhoeae* was 2.0%, *Chlamydia trachomatis* was 18.2%, and *Treponema pallidum* was 0%; only 1% of women reported being diagnosed with an STI in the past six months. Women included in qualitative interviews were not substantially different from the whole cohort.

**Table 1:** Demographic, clinical and sexual behaviour characteristics of Kenyan women participating in sexual health and PrEP willingness survey following care for a pregnancy loss

	All women Median (IQR) or N (%) N = 198	Women from Thika Median (IQR) or N (%) N = 114	Women from Kisumu Median (IQR) or N (%) N = 84	Women participating in qualitative interviews Median (IQR) or N (%) N = 30
Demographic				
Age	21 (19, 22)	21 (20, 22)	21 (19, 22)	21 (19, 21)
Education, years	13 (11, 14)	13 (11, 14)	12 (11, 14)	13 (11, 14)
Married or cohabiting with a partner	78 (39.4%)	63 (55.3%)	15 (17.9%)	3 (10.0%)
Number of living children	0 (0, 1)	0 (0, 1)	0 (0, 1)	0 (0, 0)
Partner provides financial/material support	147 (74.2%)	99 (86.8%)	48 (57.1%)	12 (40.0%)
Earns an income of her own				
No	137 (69.2%)	75 (65.8%)	62 (73.8%)	25 (83.3%)
Yes and has a steady salary	10 (5.1%)	4 (3.5%)	6 (7.1%)	0 (0.0%)
Yes and salary is not steady	51 (25.8%)	35 (30.7%)	16 (19.0%)	5 (16.7%)
VOICE risk score^a^	5 (3, 6)	4 (2, 5)	6 (4.5, 7)	6 (5, 7)
VOICE risk score^a^ >4	103 (52.0%)	40 (35.1%)	63 (75.0%)	28 (93.3%)
Clinical				
Diagnosed with an STI in the past 6 months (self‐reported)	2 (1.0%)	1 (0.9%)	1 (1.2%)	0 (0.0%)
*Chlamydia trachomatis* positive	36 (18.2%)	19 (16.7%)	17 (20.2%)	9 (30.0%)
*Neisseria gonorrhoeae* positive	4 (2.0%)	2 (1.8%)	2 (2.4%)	0 (0.0%)
*Treponema palladum* positive	0 (0.0%)	0 (0.0%)	0 (0.0%)	0 (0.0%)
Sexual behaviour				
Number of vaginal sex acts in past month	2 (0, 8)	4 (1, 10)	2 (0, 8)	1 (0, 3)
Frequency of condom use during vaginal sex in past month				
Never	105 (75.5%)	79 (84.9%)	26 (56.5%)	17 (73.9%)
Rarely	13 (9.4%)	5 (5.4%)	8 (17.4%)	3 (13.0%)
Sometimes	15 (10.8%)	7 (7.5%)	8 (17.4%)	2 (8.7%)
Often	1 (0.7%)	1 (1.1%)	0 (0.0%)	0 (0.0%)
Always	5 (3.6%)	1 (1.1%)	4 (8.7%)	1 (4.4%)
Used a condom use during most recent vaginal sex	23 (11.6%)	8 (7.0%)	15 (17.9%)	4 (13.3%)
Number of sex partners in past three months	1 (1, 1)	1 (1, 1)	1 (1, 1)	1 (1, 1)
Participant had sex with a new partner in past three months	18 (9.1%)	11 (9.6%)	7 (8.3%)	4 (13.3%)
Partner descriptions				
Reported HIV status of primary partner				
HIV‐positive	1 (0.5%)	0 (0.0%)	1 (1.2%)	0 (0.0%)
HIV‐negative	133 (67.2%)	85 (74.6%)	48 (57.1%)	18 (60.0%)
HIV status unknown by participant	64 (32.3%)	29 (25.4%)	35 (41.7%)	12 (40.0%)
Partner is taking ARVs	1 (0.5%)	0 (0.0%)	1 (1.2%)	0 (0.0%)
Participant felt she had to have sex with partner in exchange for money or gifts in the past one month	17 (12.0%)	6 (6.4%)	11 (22.9%)	2 (9.5%)
Lifestyle				
Used alcohol in the past one month				
Yes, heavy use^b^	12 (6.1%)	9 (7.9%)	3 (3.6%)	1 (3.3%)
Yes, not heavy use	12 (6.1%)	11 (9.6%)	1 (1.2%)	5 (16.7%)
No	174 (87.9%)	94 (82.5%)	80 (95.2%)	24 (80.0%)
Used cigarettes in the past one month	1 (0.5%)	0 (0.0%)	1 (1.2%)	0 (0.0%)
Used marijuana in the past one month	4 (2.0%)	4 (3.5%)	0 (0.0%)	2 (6.7%)

^a^ The modified VOICE risk score incorporates participant age, marital status, alcohol use, and whether the primary partner provides financial support and has additional partners

^b^ heavy alcohol use was determined using the Rapid Alcohol Problem Screening (RAPS) tool.

Approximately half (51.1%) of women reported using at least one measure to self‐manage their abortion, including 28.8% who used misoprostol or another drug and 6.6% who reported their manual vacuum aspiration (MVA) as the measure taken. This question also elicited responses from 5.1% of women who reported a medical situation as being responsible for their pregnancy loss (e.g. an ectopic pregnancy) (Table 2). When women provided an explanation for wanting to terminate their pregnancy, reasons included wanting to avoid missing opportunities, financial concerns, partners’ lack of desire for the pregnancy, and stigma. Three‐quarters of women had MVA at the post‐abortion clinic (92.1% in Thika and 54.8% in Kisumu) in addition to treatments for infection (50.5%), pain (95.2%) and blood loss (4.5%). Most women desired more children in the future (median children desired=3, IQR 2 to 3) and 46.5% had never used a contraceptive method in their lifetime.

**Table 2:** Experiences with recent pregnancy and family planning among Kenyan women participating in a sexual health and PrEP willingness survey following care for a pregnancy loss

	All women Median (IQR) or N (%) N = 198	Women from Thika Median (IQR) or N (%) N = 114	Women from Kisumu Median (IQR) or N (%) N = 84	Women participating in qualitative interviews Median (IQR) or N (%) N = 30
Measures taken to lose the most recent pregnancy^a^				
Nothing was used	97 (48.9%)	74 (64.9%)	23 (27.4%)	19 (63.3%)
Misoprostol or other drug	57 (28.8%)	12 (10.5%)	45 (53.6%)	11 (36.7%)
Herbs/natural remedy	0 (0.0%)	0 (0.0%)	0 (0.0%)	0 (0.0%)
Manual vacuum aspiration	13 (6.6%)	1 (0.9%)	12 (14.3%)	0 (0.0%)
Reported an ectopic pregnancy or other medical situation as responsible for the loss	10 (5.1%)	10 (8.8%)	0 (0.0%)	0 (0.0%)
Other method (includes travel, overworking, experiencing trauma, and unknown)	22 (11.1%)	17 (14.9%)	5 (6.0%)	0 (0.0%)
Reasons for deciding to lose the most recent pregnancy^a^				
No reasons/spontaneous loss	48 (24.2%)	22 (19.3%)	26 (31.0%)	7 (23.3%)
Already have the desired number of children	1 (0.5%)	0 (0.0%)	1 (1.2%)	0 (0.0%)
Partner does not want the child	24 (12.1%)	1 (0.9%)	23 (27.4%)	2 (6.7%)
Stigma of the pregnancy	21 (10.6%)	1 (0.9%)	20 (23.8%)	4 (13.3%)
Loss of opportunities if I have the child	37 (18.7%)	4 (3.5%)	33 (39.3%)	8 (26.7%)
Financial concerns about supporting the child	32 (16.2%)	3 (2.6%)	29 (34.5%)	7 (23.3%)
Other	46 (23.2%)	28 (24.6%)	18 (21.4%)	5 (16.7%)
Don't know	58 (29.3%)	58 (50.9%)	0 (0.0%)	8 (26.7%)
Services experienced from PAC^a^				
Manual vacuum aspiration	151 (76.3%)	105 (92.1%)	46 (54.8%)	22 (73.3%)
Blood transfusion	9 (4.5%)	4 (3.5%)	5 (6.0%)	1 (3.3%)
Medication for infection (antibiotics)	100 (50.5%)	31 (27.2%)	69 (82.1%)	14 (46.7%)
Medication for pain (e.g. ibuprofen, aspirin)	20 (95.2%)	16 (100%)	4 (80.0%)	1 (3.3%)
Other medication	40 (20.2%)	8 (7.0%)	32 (38.1%)	9 (30.0%)
Rehydration (IV fluids)	82 (41.4%)	56 (49.1%)	26 (31.0%)	12 (40.0%)
Children desired in the future	3 (2, 3)	3 (2, 3)	3 (2, 3)	3 (2, 3)
Contraceptive methods used ever^a^				
IUD	3 (1.5%)	1 (0.9%)	2 (2.4%)	1 (3.3%)
Implant	35 (17.7%)	15 (13.2%)	20 (23.8%)	2 (6.7%)
DMPA	45 (22.7%)	26 (22.8%)	19 (22.6%)	7 (23.3%)
Oral contraception	19 (9.6%)	15 (13.2%)	4 (4.8%)	3 (10.0%)
Emergency contraceptive pills	26 (13.1%)	26 (22.8%)	0 (0.0%)	3 (10.0%)
Natural methods, (e.g. withdrawal or rhythm method)	6 (3.0%)	5 (4.4%)	1 (1.2%)	1 (3.3%)
Other method	9 (4.5%)	3 (2.6%)	6 (7.1%)	1 (3.3%)
None	92 (46.5%)	47 (41.2%)	45 (53.6%)	16 (53.3%)

Responses are not mutually exclusive.

**Figure 1:**
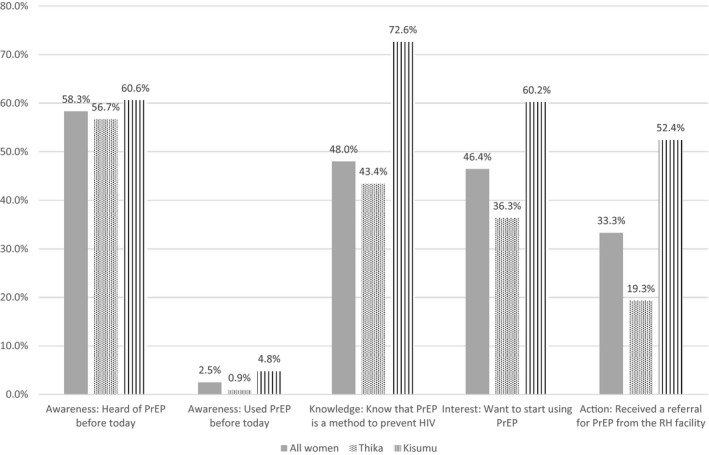
PrEP knowledge‐to‐use cascade among Kenyan women participating in a sexual health and PrEP willingness survey following care for a pregnancy loss. Women in the study had moderate knowledge and willingness to use PrEP but few had actual experience with PrEP.

Knowledge about PrEP was moderate with 58.3% of women having heard of PrEP before the day survey data were collected (56.7% in Thika and 60.6% in Kisumu) and 48.0% having knowledge that PrEP is used to prevent HIV (43.4% in Thika and 72.6% in Kisumu, Figure 1). In qualitative interviews, young women described PrEP as a tool for people who have multiple partners, partners whose HIV status is unknown or known to be positive, and even more broadly for all relationships since it is common for men to have multiple partners. In addition to preventing HIV, women described the mental health benefits, such as reducing stress.

*“She told me you take PrEP continuously if you have multiple sexual partners and you don’t know about their HIV status to prevent you from HIV or you have a partner who is [HIV]‐positive so you use PrEP so that the partner doesn’t transfer the virus to you.” (22 year old, Thika)*

*“You cannot trust these boyfriends of ours, you know like while I am here I don’t know where he is and what he is doing…maybe he is with a lady now. Yes, I can even start using it even today.” (20 year old, Thika)*

*“It prevents HIV and reduces stress because if your boyfriend says he won’t use condoms, you have already taken it so you won’t put yourself at risk." (23 year old, Thika)*

**Figure 2:**
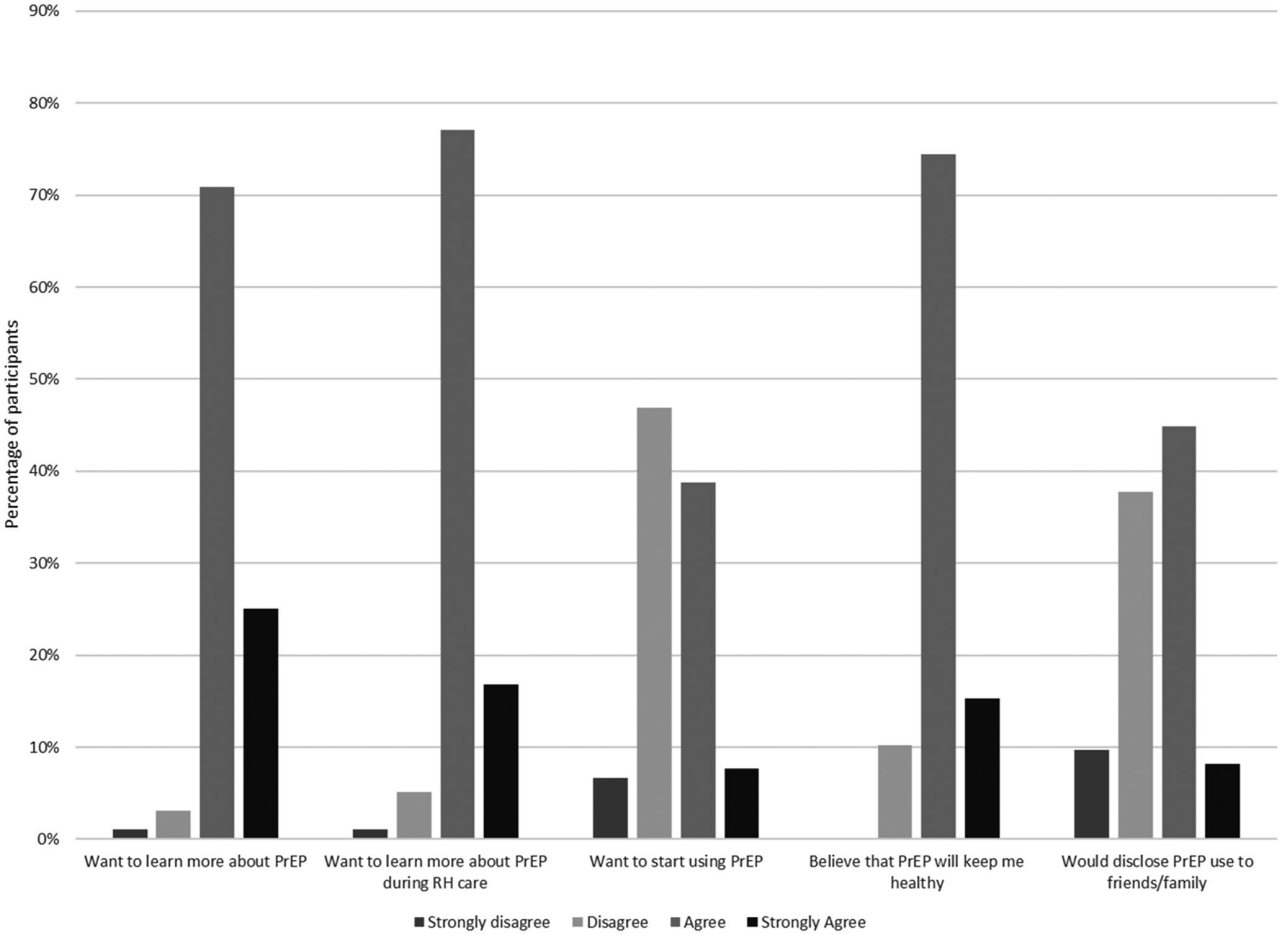
PrEP interest among Kenyan women participating in a sexual health and PrEP willingness survey following care for a pregnancy loss. Most women were in agreement or strong agreement that would want to learn more about PrEP during their reproductive health care.

Nearly half of women (46.4%) agreed that they would want to start using PrEP. All women were offered a referral and 33.3% actually accepted a PrEP referral from the study team. There were site‐specific differences with Kisumu generally having a greater frequency of knowledge and interest than Thika. In particular, 4.8% of women in Kisumu and 0.9% of women in Thika had used PrEP prior to their study participation. Women in Kisumu also had higher frequencies of wanting to start and receiving a referral (60.2% and 52.4%) than in Thika (36.3% and 19.3%). Women generally agreed or strongly agreed with statements relaying interest in PrEP (Figure 2). When probing women about how they learned about PrEP, there were some statements reflecting greater penetration of PrEP knowledge and experience into the community, such as statements about parents supporting PrEP use and learning about PrEP from a neighbour. Conversely, however, women also talked about the stigma associated with PrEP use and its relationship to HIV, indicating that these concerns are still very prevalent.

*“Most of the people are saying that PrEP is more effective than condoms because during intercourse a condom might burst and that is already a risk and PrEP, is something that is in the blood. Those who speak about this is mainly my friends and my mother. My mother usually encourages us to use PrEP rather than condoms.” (20 year old, Kisumu)*

*“I was given (PrEP) in hospital when I was admitted and that is where I was told about PrEP. I had also heard about it from my friend, who is a neighbor.” (19 year old, Thika)*

*“You know even if someone is using, they don’t want people to know, they can’t be open to tell you that they use PrEP. Maybe they think that if they tell you, you will think that they are HIV infected, the person who has no knowledge of PrEP will say that ‘so and so is infected with HIV’ because they are always similar with those drugs of HIV.” (21 year old, Kisumu)*

Among women who did not know about PrEP during qualitative interviews, some recounted feeling too tired or overwhelmed by the experience of seeking postabortion care to remember what they had been told about PrEP by the research team. While women felt that PrEP could be initiated at a post‐abortion clinic, they would prefer to seek PrEP refills from a different location, such as a family planning clinic or pharmacy, to avoid seeing people they know or recalling the negative experience of their pregnancy loss.

*“I heard about it [PrEP] but I didn’t understand about it well because at that time I was at the hospital I was tired and I didn’t get anything about it but the study nurse did her work. I would like to know more about it…she told me that when I join the program, I will get to know about it.” (21 year old, Thika)*

*“I wouldn’t like to go there [MCH or gynecology ward] and then I remember how it [pregnancy loss] happened…for me I wouldn’t want to go there.” (21 year old, Thika)*

*“They can access PrEP from there (gynae ward) but the problem would be coming back. Some fear since you may bump into someone.” (21 year old, Thika)*

Numerous characteristics were different between women who were given a PrEP referral and those who were not (Table 3). PrEP referrals were more commonly given to women who were married or cohabiting with a partner, receiving financial or material support from a partner, had a higher score on the VOICE risk score, had fewer vaginal sex acts, and used a condom during the most recent sex act. However, none of these associations were statistically significant in our multivariable model.

**Table 3:** Correlates of receiving a PrEP referral from a post‐abortion provides affiliated with the study

	N (%) receiving a PrEP referral	RR (95% CI)	*p*‐value	Adjusted RR (95% CI)	*p*‐value
Age 15 to 20	22 (41.5%)	1.36 (0.91 to 2.03)	0.31		
Age 21 to 24	44 (30.6%)	Reference			
Education ≤13 years	49 (34.5%)	1.12 (0.71 to 1.76)	0.6		
Education >13 years	17 (30.9%)	Reference			
Married/cohabiting with a partner	52 (43.7%)	2.43 (1.45 to 4.08)	0.001	1.40 (0.67 to 2.91)	0.28
Not married/cohabiting	14 (18.0%)	Reference		Reference	
Has no living children	48 (35.6%)	Reference			
Has at least 1 living child	18 (29.0%)	1.22 (0.78 to 1.92)	0.38		
Partner provides financial/material support	25 (49.0%)	1.75 (1.19 to 2.56)	0.004	1.22 (0.83 to 1.80)	0.32
Partner does not provide financial/material support	41 (28.1%)	Reference		Reference	
Earns an income of her own	16 (26.2%)	0.71 (0.44 to 1.15)	0.16		
Does not earn an income of her own	50 (36.8%)	Reference			
VOICE risk score^a^ <4	8 (15.4%)	Reference		Reference	
VOICE risk score^a^ ≥4	58 (40.0%)	2.60 (1.33 to 5.07)	0.005	1.41 (0.56 to 3.55)	0.46
Diagnosed with a bacterial^a^	11 (29.0%)	0.84 (0.49 to 1.44)	0.41		
No diagnosed STI	55 (34.6%)	Reference			
0 to 4 vaginal sex acts, past month	55 (40.4%)	Reference		Reference	
≥5 sex acts, past month	11 (18.0%)	0.45 (0.25 to 0.79)	0.006	0.72 (0.38 to 1.36)	0.31
Used a condom use during most recent vaginal sex	13 (56.5%)	1.86 (1.22 to 2.83)	0.004	1.40 (0.92 to 2.13)	0.12
Did not use a condom during most recent vaginal sex	53 (30.5%)	Reference		Reference	
Participant had sex with a new partner, past three months	6 (33.3%)	0.99 (0.50 to 1.97)	0.99		
No new partner, past three months	60 (33.5%)	Reference			
Partner is living with HIV or has status unknown to participant	26 (40.0%)	1.32 (0.89 to 1.96)	0.17		
Partner is known to be HIV negative	40 (30.3%)	Reference			
Had heavy alcohol use, past one month	6 (50.0%)	1.54 (0.84 to 2.82)	0.16		
Did not have heavy alcohol use, past one month	60 (32.4%)	Reference			

^a^ Bacterial STIs diagnosed through the study include Chlamydia trachomatis, *Neisseria gonorrhoeae*, *Treponema palladum*.

## 4 DISCUSSION

To our knowledge, these are the first data to explore knowledge and interest in HIV PrEP among Kenyan women accessing facility‐based care following an abortion. We found that these women are good candidates for PrEP based on their desire and potential for being exposed to HIV. Very few women had actual experience with PrEP; many learned about it for the first time from our research staff following their survey and one‐third received a referral to a PrEP clinic. In recent family planning and maternal and child health PrEP programmes in Kenya, approximately 20% of women have received a PrEP referral, and so women seeking post‐abortion care may accept a PrEP referral with similar or slightly greater frequency, an estimate that could be used for policy and programme planning [15]. The overall results from our study suggest that post‐abortion care and safe abortion clinics, important components of reproductive health programming, can be incorporated into policy guidance and programmes that aim to link and provide direct services for PrEP and other HIV and STI strategies.

Women’s mental health varies dramatically after an induced abortion often with changes in depression, anxiety, and substance use [26, 27]. Women in our study were willing to talk with clinic providers and our study staff about their potential for HIV exposure and to realize different methods for HIV prevention. An important key finding from the qualitative data is that women expressed a strong desire to avoid the post‐abortion clinic in the future. Thus, women who initiate PrEP through post‐abortion clinics would need strong linkage to alternative PrEP programmes, such as integrated family planning clinics, in order to receive refills and accompanying HIV testing [2, 5]. In addition, programmes that feature caring and friendly providers whose attitudes are not judgmental are better suited to younger women [28] and community‐based PrEP education services could support young women with unanswered questions regarding PrEP and HIV/STI prevention.

We found higher frequencies of PrEP knowledge and interest in women in Kisumu than Thika. HIV incidence in Kisumu is the highest in Kenya and this region has been scaling up PrEP and other prevention interventions very quickly in the past five years [29, 30, 31]. Thus, it is likely that community awareness of PrEP and normalization for using HIV prevention has penetrated further into the community. This penetration has been cited by women in South Africa for providing social support and normalizing PrEP use and it is likely to foster opportunities for PrEP provision at a wide range of health clinics and facilities, including post‐abortion clinics [32].

This formative study was designed to inform a PrEP delivery programme that will be integrated into existing post‐abortion clinics in Kenya and to begin developing rapport for research to be conducted in these settings. Thus, our study is limited in size, we were not actually providing PrEP to study participants, and we do not have data on whether PrEP referrals were realized by participants. Additionally, our study is based on sequentially recruiting women accessing post‐abortion care at health facilities, which is somewhat of a convenience sample and misses a large segment of women who induce abortion at home and do not need to seek medical care. Since abortion and seeking post‐abortion care is highly stigmatized, there are no data to determine if our sample is representative of all women who seek post‐abortion care. Our efforts to talk to pharmacists that provide medical abortion pills did not yield methods to include them in this study but these are an important source to learn from and consider as PrEP providers in future work [33]. A final limitation is that we targeted our in‐depth interview sampling to women with high risk as determined through a tool developed and validated with women from South Africa. Since women in our study were Kenyan, the tool may not be fully applicable, although one‐fifth of the women in our study were infected with *Chlamydia trachomatis* or *Neisseria gonorrhoeae,* and thus likely at substantial risk for HIV.

## 5 CONCLUSIONS

Post‐abortion care is an existing service that can be better leveraged to integrate HIV and STI services and other facets of reproductive health (e.g. HPV screening and vaccination, postpartum family planning). An added benefit of integration is the opportunity to reduce the stigma associated with attending a post‐abortion clinic since multiple services are available. Women in this study expressed openness to receiving these types of services and engaging with comprehensive services once their immediate needs related to pregnancy loss were met. To advance the delivery of comprehensive sexual and reproductive health services, post‐abortion clinics can become a component of national programmes for PrEP and other HIV and STI services.

## Authors’ contributions

RH, NM and TB conceived the study design. EC, LA, BK, VO, CK, FA, NW, EO, HM, CS and EB conducted data collection. KN, DT, BK, EC, VO and YZ conducted data analysis. RH and NM prepared the first draft of the paper. All authors read and approved the final manuscript.

## Funding

This work was supported by Children’s Investment Fund Foundation (R‐1808‐02992).

## References

[1] Lindegren ML , Kennedy CE , Bain‐Brickley D , Azman H , Creanga AA , Butler LM , et al. Integration of HIV/AIDS services with maternal, neonatal and child health, nutrition, and family planning services. Cochrane Database Syst Rev. 2012:CD010119. 22972150 10.1002/14651858.CD010119 PMC12551653

[2] Mugwanya KK , Pintye J , Kinuthia J , Abuna F , Lagat H , Begnel ER , et al. Integrating preexposure prophylaxis delivery in routine family planning clinics: A feasibility programmatic evaluation in Kenya. PLoS Med. 2019;16:e1002885. 31479452 10.1371/journal.pmed.1002885 PMC6719826

[3] Spaulding AB , Brickley DB , Kennedy C , Almers L , Packel L , Mirjahangir J , et al. Linking family planning with HIV/AIDS interventions: a systematic review of the evidence. AIDS (London, England). 2009;23 Suppl 1:S79–88. 10.1097/01.aids.0000363780.42956.ff 20081392

[4] Buzi RS , Madanay FL , Smith PB . Integrating routine HIV testing into family planning clinics that treat adolescents and young adults. Public Health Rep. 2016;131 1_suppl:130–138. 26862238 10.1177/00333549161310S115 PMC4720614

[5] Kinuthia J , Pintye J , Abuna F , Mugwanya KK , Lagat H , Onyango D , et al. Pre‐exposure prophylaxis uptake and early continuation among pregnant and post‐partum women within maternal and child health clinics in Kenya: results from an implementation programme. Lancet HIV. 2020;7(1):e38–48. 31813837 10.1016/S2352-3018(19)30335-2 PMC11498332

[6] Heffron R , Ngure K , Velloza J , Kiptinness C , Quame‐Amalgo J , Oluch L , et al. Implementation of a comprehensive safer conception intervention for HIV‐serodiscordant couples in Kenya: uptake, use and effectiveness. J Int AIDS Soc. 2019;22:e25261. 30957420 10.1002/jia2.25261 PMC6452026

[7] Schwartz SR , Bassett J , Mutunga L , Yende N , Mudavanhu M , Phofa R , et al. HIV incidence, pregnancy, and implementation outcomes from the Sakh'umndeni safer conception project in South Africa: a prospective cohort study. Lancet HIV. 2019;6(7):e438–46. 31160268 10.1016/S2352-3018(19)30144-4 PMC6660144

[8] Ministry of Health, National AIDS & STI Control Program . Guidelines on use of antiretroviral drugs for treating and preventing HIV infection in Kenya 2018 Edition. Nairobi, Kenya; 2018.

[9] Eakle R , Gomez GB , Naicker N , Bothma R , Mbogua J , Cabrera Escobar MA , et al. HIV pre‐exposure prophylaxis and early antiretroviral treatment among female sex workers in South Africa: Results from a prospective observational demonstration project. PLoS Med. 2017;14:e1002444. 29161256 10.1371/journal.pmed.1002444 PMC5697804

[10] Morton J , Bukusi E , Delaney‐Moretlwe S , Bekker LG , Omollo V , Travill D , et al. High prevalence of curable STIs among young women initiating PrEP in Kenya and South Africa. Abstract #WEPEC223. AIDS 2018; Amsterdam, Netherlands; 2018.

[11] Mohamed SF , Izugbara C , Moore AM , Mutua M , Kimani‐Murage EW , Ziraba AK , et al. The estimated incidence of induced abortion in Kenya: a cross‐sectional study. BMC Pregnancy Childbirth. 2015;15:185. 26294220 10.1186/s12884-015-0621-1 PMC4546129

[12] Makenzius M , Faxelid E , Gemzell‐Danielsson K , Odero TMA , Klingberg‐Allvin M , Oguttu M . Contraceptive uptake in post abortion care‐Secondary outcomes from a randomised controlled trial, Kisumu, Kenya. PLoS One. 2018;13:e0201214. 30096148 10.1371/journal.pone.0201214 PMC6086397

[13] Rasch V , Yambesi F , Massawe S . Post‐abortion care and voluntary HIV counselling and testing–an example of integrating HIV prevention into reproductive health services. Trop Med Int Health. 2006;11(5):697–704. 16640622 10.1111/j.1365-3156.2006.01607.x

[14] Kabiru CW , Ushie BA , Mutua MM , Izugbara CO . Previous induced abortion among young women seeking abortion‐related care in Kenya: a cross‐sectional analysis. BMC Pregnancy Childbirth. 2016;16:104. 27180102 10.1186/s12884-016-0894-z PMC4867193

[15] Ministry of Health (MOH) . K4Health. Introduction to PAC service delivery guidelines. 2011. [cited 2016 Dec 11]. Accessed from: http://www.postabortioncare.org/content/introduction‐pac‐service‐delivery‐guidelines

[16] Center for Reproductive Rights . World Abortion Laws. 2016. Accessed from: http://worldabortionlaws.com/map/

[17] Gebreselassie H , Gallo MF , Monyo A , Johnson BR . The magnitude of abortion complications in Kenya. . BJOG Int J Obstetr Gynaecol. 2005;112(9):1229–35. 10.1111/j.1471-0528.2004.00503.x 16101601

[18] Tavrow P , Withers M , McMullen K . Age matters: differential impact of service quality on contraceptive uptake among post‐abortion clients in Kenya. Culture, Health Sex. 2012;14(8):849–62. 10.1080/13691058.2012.700324 22812449

[19] Benson J , Andersen K , Brahmi D , Healy J , Mark A , Ajode A , et al. What contraception do women use after abortion? An analysis of 319,385 cases from eight countries. Glob Public Health. 2016;13:1–16. 27193827 10.1080/17441692.2016.1174280

[20] Cherpitel CJ , Ye Y , Bond J , Borges G , Cremonte M , Marais S , et al. Cross‐national performance of the RAPS4/RAPS4‐QF for tolerance and heavy drinking: data from 13 countries. J Stud Alcohol. 2005;66(3):428–32. 16047534 10.15288/jsa.2005.66.428

[21] Evidence for Contraceptive Options and HIV Outcomes (ECHO) Trial Consortium . HIV incidence among women using intramuscular depot medroxyprogesterone acetate, a copper intrauterine device, or a levonorgestrel implant for contraception: a randomised, multicentre, open‐label trial. Lancet. 2019;394(10195):303–13. 31204114 10.1016/S0140-6736(19)31288-7 PMC6675739

[22] Baeten JM , Donnell D , Ndase P , Mugo NR , Campbell JD , Wangisi J , et al. Antiretroviral prophylaxis for HIV prevention in heterosexual men and women. N Engl J Med. 2012;367(5):399–410. 22784037 10.1056/NEJMoa1108524 PMC3770474

[23] Sandelowski M . Sample size in qualitative research. Res Nurs Health. 1995;18(2):179–83. 7899572 10.1002/nur.4770180211

[24] Balkus J , Zhang J , Nair G , Palanee T , Ramjee G , Nakabilito C , et al. Development of a risk scoring tool to predict HIV‐1 acquisition in African women. AIDS Research Human Retroviruses. 2014;30(S1):A214.

[25] Azungah T . Qualitative research: deductive and inductive approaches to data analysis. Qualitative Research Journal. 2018;18(4):383–400.

[26] Horvath S , Schreiber CA . Unintended pregnancy, induced abortion, and mental health. Curr Psychiatry Rep. 2017;19(11):77. 28905259 10.1007/s11920-017-0832-4

[27] Major B , Cozzarelli C , Cooper ML , Zubek J , Richards C , Wilhite M , et al. Psychological responses of women after first‐trimester abortion. Arch Gen Psychiatry. 2000;57(8):777–84. 10920466 10.1001/archpsyc.57.8.777

[28] Denno DM , Hoopes AJ , Chandra‐Mouli V . Effective strategies to provide adolescent sexual and reproductive health services and to increase demand and community support. J Adolescent Health, 2015;56 1 Suppl:S22–41. 10.1016/j.jadohealth.2014.09.012 25528977

[29] Mugwanya KK , Irungu E , Bukusi E , Mugo NR , Odoyo J , Wamoni E , et al. Scale up of PrEP integrated in public health HIV care clinics: a protocol for a stepped‐wedge cluster‐randomized rollout in Kenya. Implement Sci. 2018;13(1):118. 30180860 10.1186/s13012-018-0809-7 PMC6123996

[30] Masyuko S , Mukui I , Njathi O , Kimani M , Oluoch P , Wamicwe J , et al. Pre‐exposure prophylaxis rollout in a national public sector program: the Kenyan case study. Sexual health. 2018;15(6):578–86. 30408432 10.1071/SH18090 PMC7206896

[31] Were D , Musau A , Mutegi J , Ongwen P , Manguro G , Kamau M , et al. Using a HIV prevention cascade for identifying missed opportunities in PrEP delivery in Kenya: results from a programmatic surveillance study. J Int AIDS Soc. 2020;23:e25537‐e. 32602658 10.1002/jia2.25537 PMC7325512

[32] Velloza J , Khoza N , Scorgie F , Chitukuta M , Mutero P , Mutiti K , et al. The influence of HIV‐related stigma on PrEP disclosure and adherence among adolescent girls and young women in HPTN 082: a qualitative study. J Int AIDS Soc. 2020;23:e25463. 32144874 10.1002/jia2.25463 PMC7060297

[33] Tung EL , Thomas A , Eichner A , Shalit P . Implementation of a community pharmacy‐based pre‐exposure prophylaxis service: a novel model for pre‐exposure prophylaxis care. Sexual Health. 2018;15(6):556–61. 30401342 10.1071/SH18084

